# An Improved Protocol for Sequencing of Repetitive Genomic Regions and Structural Variations Using Mutagenesis and Next Generation Sequencing

**DOI:** 10.1371/journal.pone.0043359

**Published:** 2012-08-17

**Authors:** Botond Sipos, Tim Massingham, Adrian M. Stütz, Nick Goldman

**Affiliations:** 1 European Bioinformatics Institute (EMBL-EBI), Wellcome Trust Genome Campus, Hinxton, Cambridge, United Kingdom; 2 European Molecular Biology Laboratory (EMBL), Genome Biology Research Unit, Heidelberg, Germany; University of Illinois at Chicago, United States of America

## Abstract

The rise of Next Generation Sequencing (NGS) technologies has transformed *de novo* genome sequencing into an accessible research tool, but obtaining high quality eukaryotic genome assemblies remains a challenge, mostly due to the abundance of repetitive elements. These also make it difficult to study nucleotide polymorphism in repetitive regions, including certain types of structural variations. One solution proposed for resolving such regions is Sequence Assembly aided by Mutagenesis (SAM), which relies on the fact that introducing enough random mutations breaks the repetitive structure, making assembly possible. Sequencing many different mutated copies permits the sequence of the repetitive region to be inferred by consensus methods. However, this approach relies on molecular cloning in order to isolate and amplify individual mutant copies, making it hard to scale-up the approach for use in conjunction with high-throughput sequencing technologies. To address this problem, we propose NG-SAM, a modified version of the SAM protocol that relies on PCR and dilution steps only, coupled to a NGS workflow. NG-SAM therefore has the potential to be scaled-up, e.g. using emerging microfluidics technologies. We built a realistic simulation pipeline to study the feasibility of NG-SAM, and our results suggest that under appropriate experimental conditions the approach might be successfully put into practice. Moreover, our simulations suggest that NG-SAM is capable of reconstructing robustly a wide range of potential target sequences of varying lengths and repetitive structures.

## Introduction

Thanks to the increased throughput provided by Next Generation Sequencing technologies [1], *de novo* genome sequencing and resequencing are now widely accessible research tools, significantly contributing to the advancement of many fields of biology and with many important applications. However, at least in the case of second generation technologies, the length of the obtained reads is below that provided by “traditional" Sanger sequencing. Read length is critical for obtaining high-quality genome assemblies, as longer reads are more likely to capture the context of repetitive units (see later). For this reason, genome assembly is more difficult when using NGS technologies [2], [3] and so, despite the increase in sequencing throughput, obtaining “finished" assemblies of eukaryotic genomes remains a challenge that requires laborious experiments to resolve the problematic regions on a case-by case basis [4], [5].

**Figure 1:**
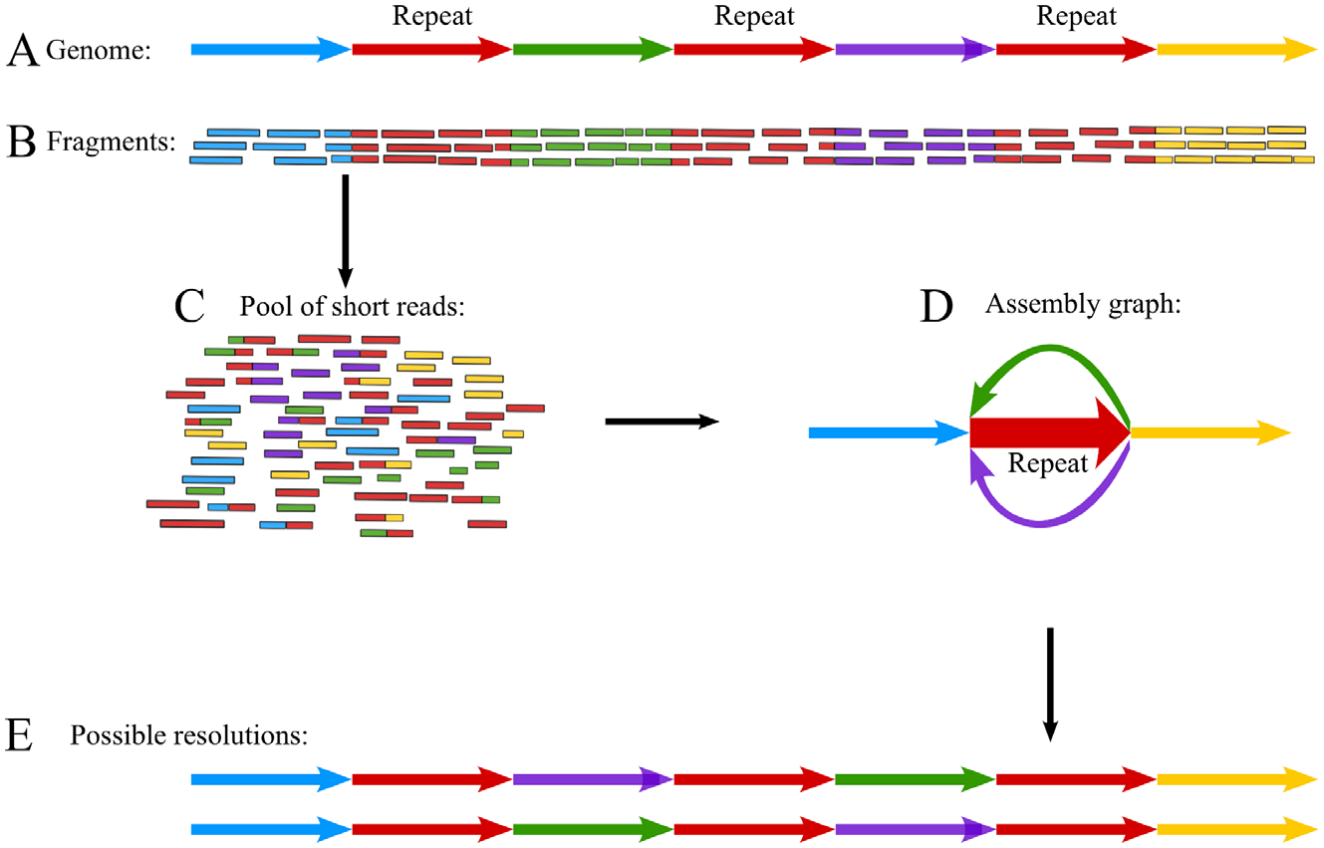
Assembly problems caused by the presence of repeats. **A**. The structure of the target region. Red units are identical or near-identical; other colours are unique. **B**. Fragments ordered by their origin. **C**. Pool of reads obtained by short read sequencing. Note that in this example the full length of the fragments is sequenced. **D**. A graph structure summarizing assembly uncertainty. The thickness of the arrows representing the units is indicative of the depth of coverage. **E**. The two possible resolutions of the assembly graph, given that the copy numbers of all of the units are estimated correctly.

One of the main causes of assembly difficulties is the structure of the eukaryotic genome itself, and more precisely the abundance of repetitive elements (e.g. transposons), which leads to fragmented assemblies or complex misassemblies depending on the approach taken by the assembler [2], [3]. Moreover, repetitive regions are frequently structural variation hotspots [6], further complicating their assignment and assembly. We illustrate the assembly problems caused by repeats by a hypothetical example targeting the assembly of the genomic region with the structure shown in Figure 1A. The region has seven units, four being unique (light blue, green, dark blue and yellow units), and three being copies of the same unit (red). The red units can be considered to be completely identical, but this is not necessary for assembly problems to present themselves.

The pool of fragments from which the sequenced reads originate (the sequencing “library") is produced by random fragmentation of many identical copies of the target region. NGS sequencing of the target region provides a set of short reads covering it multiple times (Figure 1B). The reads are obtained by sequencing a fixed number of consecutive bases from one or both ends of fragments sampled from the library. Sequencing both ends of the fragments (“paired-end sequencing", [2]) yields “read pairs", which in combination with the distribution of the fragment lengths are informative about the relative position and orientation of the two reads in the genome of origin.

During the sequencing procedure, each read loses all information about its exact position and orientation (Figure 1C). However, some reads span more than one unit and these reads are exploited by assembly methods to reconstruct the order and orientation of the units [3] and hence the structure of the genomic region. There is a variety of assembly methods using different algorithms and data structures to assemble reads into contigs [3], [7]–[9] but they are all conceptually based on detecting overlaps between reads.

In the case of regions harbouring repetitive units that are longer than the read length, no read can capture flanking sequence from both sides of a repeat unit. For this reason, read overlaps do not confer enough information on the context of the repetitive units to allow the recovery of their exact order and orientation [2], [3]. In these cases, the output of the assembly is usually a graph structure summarizing the uncertainty about the structure of the assembled region (Figure 1D). Even if the copy number of the repetitive unit is estimated from the multiplicity (depth) of coverage, the exact order and orientation will remain unknown as the assembly graph has multiple conflicting resolutions (Figure 1E). In the absence of further information, such as a physical map or longer reads, the assembly graph is left unresolved and it is broken down to contiguous sequence stretches (“contigs"). Problematic regions like this can be created by tandem gene duplications or repeated insertions of transposons, events that occur relatively frequently during the evolution of eukaryotic genomes [10], [11].

Repetitive regions can also cause problems in the context of genome resequencing as well, when the goal is not the assembly of the genome itself but the assessment of the differences compared to a known “reference" genome sequence [12]. For example, consider the simple case of a young tandem duplication: after aligning the reads back to the reference genome (“mapping"), the number and orientation of the units might be inferred based on the paired-end end signatures (the orientation of the mapped reads in a pair) and the depth of coverage [13]. However, when the units are very similar to each other, there is no hope of reconstructing their exact sequence by short read sequencing, as the reads cannot be assigned to their originating units. This makes it very difficult to study natural variation in these regions. A notable case where this issue might hinder the in-depth study of SNPs (single nucleotide polymorphisms) inside duplicated regions by short read sequencing is the CCL3L1 gene, which modulates susceptibility to HIV infection and progression to AIDS, and is highly variable in copy number due to a hot-spot of segmental duplications [14], [15].

There are techniques to improve the quality of the assemblies of repetitive regions [2]. One of them is to obtain longer reads, which is subject to budget trade-offs and strongly depends on the development of sequencing technologies. The other is to use paired-end sequencing with (possibly multiple) libraries of tight fragment size distributions, which can be exploited by advanced assembly methods to resolve repetitive regions that are not significantly longer than the fragment length [16]. Both of these approaches involve significant cost and are limited by the feasibility of the library construction step.

A conceptually elegant and simple alternative approach proposed by Keith et al. [17], [18], known as “Sequence Assembly aided by Mutagenesis" (SAM), can in principle solve the problems described above. The essence of the strategy is to sequence randomly mutated copies of the original problematic region. Introducing enough mutations eliminates the undesirable features of the target region (e.g. repetitiveness). The original sequence information is not destroyed, it is just distributed between the variants generated via mutagenesis, and hence the original sequence can be inferred by consensus [17]. In other words, if the number of random mutations introduced is high enough, then individual mutated copies are no longer repetitive and can be assembled without any ambiguity. After sequencing many independently mutated copies, the original sequence can be reconstructed by aligning the mutant sequences and calling a consensus using a simple voting scheme [19] or more advanced probabilistic algorithms [20], . The accuracy of the reconstruction can be increased by simply generating and sequencing additional mutants. Keith et al. [21] provide a way to calculate the number of mutants needed to achieve a desired accuracy given the characteristics of the mutation process.

Keith et al. successfully used the SAM approach in a proof-of-concept wetlab study [17] using Sanger sequencing technology on regions that were otherwise difficult to sequence. In a later paper, they used a computer simulation to explore the usefulness of the SAM approach when combined with short read rather than Sanger sequencing [19], including the simulated assembly of a complete *Mycoplasma genitalium* genome (0.58 Mbp). Based on the results of their simulations, Keith et al. concluded that the SAM approach combined with NGS technologies could be more cost effective than traditional approaches. They also developed advanced algorithms for reconstructing the target sequence from the mutated sequences, even taking into account the effects of sequence alignment uncertainty [21].

The crucial steps in the original SAM protocol are the amplification of the target genomic region using a mutagenic PCR protocol and Sanger sequencing of the generated variants. The detailed experimental steps needed for the SAM protocol can be summarized as follows: use mutagenic PCR relying on special bases (base analogues [22] available as a commercial kit [23]) to introduce mutations; clone the products and isolate plasmids from individual colonies; use the isolated plasmids as templates for Sanger sequencing [17]. The mutagenic PCR protocol itself consists of two rounds of amplification and a dilution/sampling step. The first PCR is performed in the presence of dNTPs with base analogues serving the purpose of introducing mutations [22]. The goal of the second “cleanup" PCR, performed without special dNTPs added, is to remove the base analogues which might cause problems during the downstream experiments.

It is important to note that the SAM approach requires independently mutated copies in order to be able to call an unbiased consensus. The product of the mutagenic PCR is a population of potentially unique mutant molecules, but many of their mutations are related by their ancestry. However, under realistic experimental protocols, the high number of starting molecules relative to the number of the sampled molecules makes it very unlikely that sampled mutants will carry mutations sharing common ancestry [17].

The original SAM protocol makes use of molecular cloning [17] in order to isolate and amplify individual mutant types serving as template for sequencing. Cloning is a laborious and costly procedure and this makes it impractical to use the SAM approach with NGS technologies, which have been enabled by high-throughput “clonal" amplification in the absence of a vector or live cell.

We propose a modified SAM protocol, NG-SAM (for “NGS-coupled SAM"), that omits the cloning step and relies solely on PCR and dilution and so has considerably more potential for scaling up. We demonstrate the feasibility of this approach using realistic simulation of the protocol and provide an analysis of the benefits and shortcomings of the SAM approach.

### The NG-SAM Protocol

**Figure 2:**
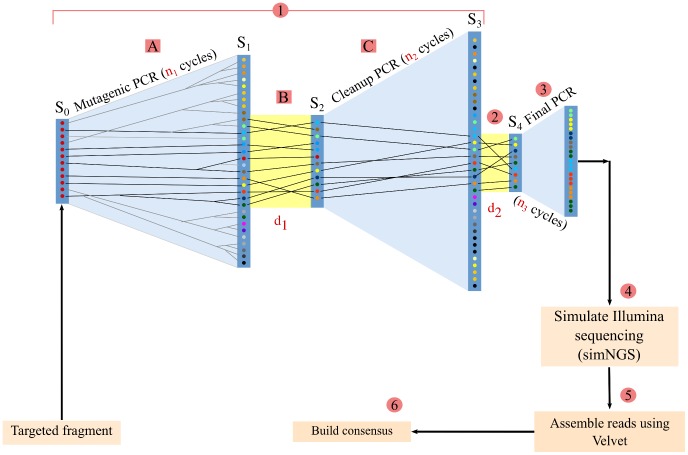
Overview of the simulated NG-SAM protocol. The numbering corresponds to the steps enumerated above in the main text. The trapezoids shaded in light blue represent PCR amplifications (with 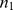
–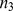 being the number of cycles), while the rectangles shaded in yellow represent sampling of molecules by dilution. 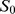
–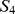 are the number of molecules present in the various stages of the simulated experiment, with unique variants symbolised by different coloured dots. 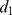 and 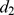 are the dilution factors corresponding to the first and second dilution steps. The black lines represent the “lineages" of the molecules sampled by the second dilution, traced back to the initial molecule pool of size 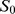. The steps 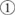 **A**–**C** correspond to the mutagenic PCR, dilution and cleanup PCR steps of the mutagenic protocol. simNGS [35] is a software for simulating Illumina sequencing and Velvet [8] is a short read assembler.

The protocol we propose, NG-SAM, can be summarized as follows:

1. Use a mutagenic PCR protocol [23], similar to the original SAM approach, in order to generate mutated copies of the target region. The mutagenic PCR protocol itself involves two PCRs: a mutagenic one (step 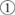 **A** in Figure 2) and a normal, cleanup PCR (step 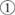 **C** in Figure 2). It also requires a dilution of the product of the mutagenic PCR (step 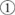 **B** in Figure 2).

Applying this mutagenic protocol is necessary to ensure the success of the short read assembly by making the target sequence less repetitive.

1. Reduce the number of mutant types present in the sample by diluting the product of the mutagenic PCR protocol.
2. Perform a final, normal PCR to amplify the remaining individual mutant molecules and then fragment to create a library with the coverage necessary for *de novo* assembly. Note that the use of proof-reading polymerase is not strictly necessary at this stage, as the errors introduced in the late PCR cycles would be simply discarded during the assembly process, while early errors will be corrected when calling the consensus.
3. Sequence the library using a standard NGS sequencing protocol.
4. Perform short read assembly, resulting in distinct DNA segments (contigs) for each mutant present in the template.
5. Align the contigs and reconstruct the target region by calling a “majority-vote" consensus (or any other effective method).

The NG-SAM approach uses dilution and PCR amplification to obtain many copies of an individual mutant type. This is fairly similar to the original SAM protocol, which implicitly uses dilution to sample mutant types during the molecular cloning step.

It should be noted that direct short read sequencing of the products of the mutagenic PCR protocol is not sufficient even after dilution: every mutant molecule is present in only one copy and we would not have the coverage necessary for *de novo* assembly, which can be created only by fragmenting a large pool of identical molecules. After performing a third PCR, the assembly of the pooled mutant types is possible, although this problem is harder than assembling a single mutant as it is necessary for every repeated unit present in the mixture (each repeat of each mutant type) to be distinguishable following the mutagenic process.

## Results

We simulated experiments in two different settings, each aiming to explore different properties of NG-SAM. The goal of the first simulation setting was to study whether a single, well-tuned set of experimental conditions is able to reconstruct a wide range of target sequences. The second simulation studied the robustness of NG-SAM to fluctuations in the dilution factors, parameters that are the most likely to influence the success of a wetlab NG-SAM experiment.

An NG-SAM experiment has many parameters to be tuned, the most important being 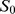, the number of starting molecules, the dilution factors 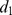 and 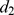 – parameters that in combination with the PCR efficiencies ultimately determine the distribution of the number of mutant types in the sequenced mixture – and the number of mutations introduced by the mutagenic PCR (Figure 2; see also Materials and Methods). Appropriate values are needed in order to prevent assembly failure by reducing the number of mutant types, while still retaining enough of them in order to obtain high accuracy after consensus calling.

Instead of trying to calculate exactly the number of molecules in the sample generated by the NG-SAM protocol, we approximated it by using the expected number of molecules after each experimental step as input for the next step. For example, in order to approximate the number of molecules after the first dilution we took the expected number of molecules after the first PCR (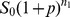, 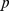 being the amplification efficiency) and divided it by the first dilution factor (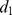). Given values of 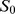, the numbers 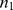
–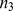 of PCR cycles and the amplification efficiencies, this approximation makes it possible to tune 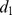 and 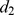 and obtain a number of mutant types close to a desired value. However, the variance of the distribution of the mutant types cannot be controlled independently from the expected value and it is also hard to predict the optimal number of mutant types for a given mutation intensity and target region. As a consequence, the parameters of the protocol may still require further tuning via simulations and trial and error.

The first setting consists of simulated NG-SAM experiments (Figure 2) targeting a range of sequences with varying length and repetitive structure under a fixed set of experimental conditions. The target sequences used in the simulations were created by concatenating randomly generated sequence units with lengths ranging from 4 up to 4,000 (step size 5). The number of repetitive units varied from 4 up to 100 (step size 4). Five target sequences were generated for each unit length/unit number combination if the total length was less or equal than 30 kb and independent experiments were simulated for each.

We used the tuning approach described above to choose parameters for our simulations. The most important ones are summarized in Table 1 (for the other parameters see the Materials and Methods section). The expected number of sites mutated was approximately 10%, as extrapolated from the calibration of the mutation process (see in the Materials and Methods section). We believe that this mutation rate should be sufficient to enable the reconstruction of most target regions of size 10 kb.

**Table 1:** The chosen values of the most important parameters used in the first simulation setting.

Name	Parameter	Value
*S*_0_	No. initial molecules	5,000
*n*_1_	No. cycles – mutagenic PCR	20
*d*_1_	Dilution factor – first dilution	70,000
*n*_2_	No. cycles – cleanup PCR	20
*d*_2_	Dilution factor – second dilution	16×10^6^
*n*_3_	No. cycles – final PCR	30

See Figure 2 for a summary of the NG-SAM protocol.

The simulated experiments can fail at various stages, either during the simulation of the NG-SAM protocol itself (e.g. if there are no molecules left after the second dilution), or during the processing of simulated reads. We consider a simulated experiment to be “successful" if no failures occurred and the called consensus sequence aligned unambiguously to the original target sequence; hence the percentage of recovered sequence length and identity can be calculated. The percentage of successful simulations turned out to be the most informative metric to assess the performance of NG-SAM under different conditions. For the successful experiments, the percentage of recovered sequence length and the percentage identity with respect to the target sequence provide further metrics for evaluating performance.

In the first simulation setting all but a few failures were caused by the inability to determine the strandedness of the assembled contigs relative to the longest contig, meaning that the failed experiments never reached the multiple alignment stage.

The distribution of the number of mutant types (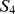 in Figure 2) from all successful simulated experiments – influenced by virtually all parameters in the simulation – had a mean of 21.79 and a standard deviation of 4.16 (Figure S1). The moderate mean and the relatively low variance of this distribution indicates expected good performance for recovering the sequence of the target region.

**Figure 3:**
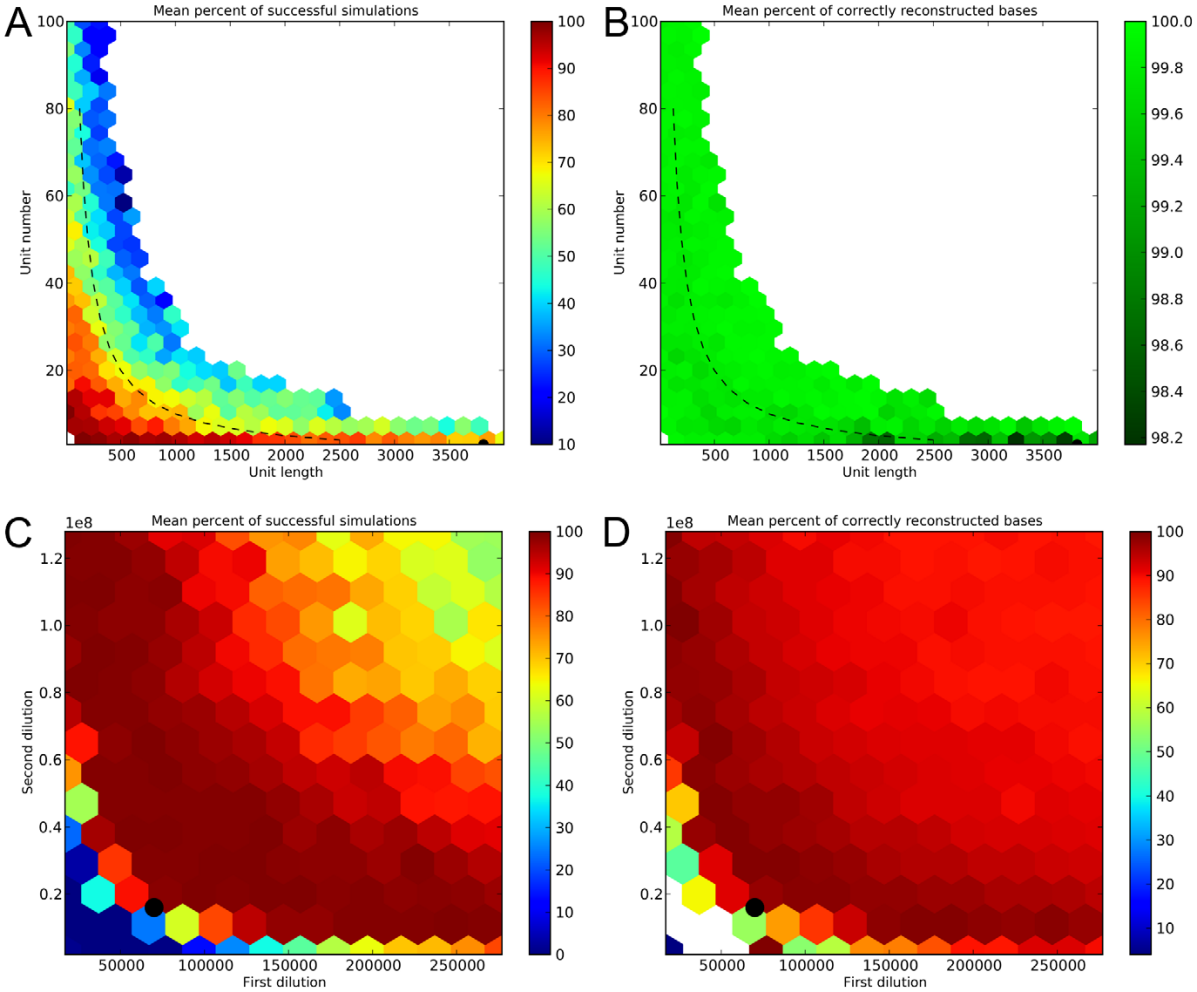
Performance of NG-SAM in simulated experiments. The hexagons are colored according to the mean of the metrics from all covered simulated experiments. White areas represent unexplored parameter space. **A**. The percentage of successful simulated experiments in the first simulation setting, as a function of length and number of repetitive units. The black circle [at the point (3813, 3)] marks the repetitive structure of the target region used in the second simulation setting. The dashed line corresponds to target regions with a total size of 10 kb. **B**. Percentage of correctly reconstructed bases in the successful experiments from the first simulation setting, as a function of length and number of repetitive units in the target sequence (black circle and dashed line as in **A**). **C**. The percentage of successful simulated experiments in the second simulation setting, as a function of the dilution factors (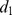 and 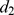 in Figure 2). The black circle corresponds to the dilution factors used in the first simulation setting. **D**. Percentage of correctly reconstructed bases in the second simulation setting as a function of the dilution factors. Black circle as in **C**; see text for further details.

The results of the first simulated settings showed that even under the single set of experimental conditions used, there is a “safe" space of unit number/length combinations in which the great majority of simulated NG-SAM experiments succeed (red/orange region, Figure 3A). This safe region includes all target sequences with a length less than or equal to the maximum length which we believe can be handled in an actual wetlab NG-SAM experiment (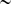
10 kb). It is also worth mentioning that the safe region also includes the target sequences having four units with the length in the range 1,000–4,000 bp, which means that the approach has indeed the potential to sequence young tandem gene duplications. From the percentage of correctly reconstructed bases (a product of percent sequence identity with respect to the target sequence – Figure S2C – and proportion of recovered target sequence length – Figure S2D) shown in Figure 3B we can conclude that the accuracy of reconstruction is high for all successful simulated experiments. More importantly, the percentage identity of the aligned portion of the called consensus sequences as compared to the target sequence is always higher than 99%.

The second simulation setting aimed to study the robustness of NG-SAM to fluctuations in the dilution factors, the most important experimental parameters determining success. Values for the first dilution factor (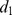) were taken across the range 17,000–280,000 (step size 20,000), and the values for the second dilution factor (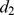) were taken across the range 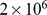
–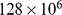 (step size 200,000). For each combination of dilution factors, five independent experiments were simulated with the rest of the parameters set identically to the ones used in the first simulation setting (Table 1).

In this second simulation setup we used as a target sequence three concatenated copies of the sequence of the *eater* gene of *Drosophila melanogaster*, without any “linker" sequences. The *eater* gene itself has a repetitive structure [24], [25] due to the presence of domain repeats (Figure S3). The length of one unit is 3,813 base pairs, so the length of the target region was 11,439 base pairs. A single mutation was introduced at a random position in every repeat unit for tracking purposes. The presence of this mutation did not make the assembly appreciably easier, but facilitated the assessment of whether the repetitive units were recovered in the correct order and orientation.

The simulations suggest that NG-SAM is relatively robust, as there is a large region close to the dilution factors chosen for the first simulation setting in which the great majority of the simulated experiments succeeded (Figure 3C). The “safe region" is approximately delineated by the product of the two dilution factors: if the overall dilution is not high enough, assembly is likely to fail (bottom left corner), on the other hand if the overall dilution is too extreme, the experiments might fail because too few molecules are sampled (upper right corner).

The safe region coincides with experiments recovering the target region with high accuracy in terms of percentage correctly reconstructed bases (Figure 3D; cf. Figure 3C). This metric shows a similar behaviour to the percentage of successful simulations in the case of experiments with extreme (too low or too high) overall dilutions. However, in this case we can interpret this behaviour as the superposition of opposing trends in terms of percentage identity and percentage reconstructed length. For successful simulations, the percentage identity is decreasing as the overall dilution increases (Figure S4C), which is most likely due to the decreasing number of mutant types used for calling the consensus. On the other hand, the percentage reconstructed sequence length for successful simulations remains high as the overall dilution increases (Figure S4D), as potentially even a single mutant type is enough to recover the length of the target region.

## Discussion

Despite constantly–increasing sequencing throughput, obtaining high-quality eukaryotic genome assemblies remains a challenge due to the presence of repetitive elements longer than the reads provided by second generation sequencing technologies. A promising approach pioneered by Keith et al. [17], [21] known as “Sequence Assembly aided by Mutagenesis" (SAM) exploits the fact that the sequencing of highly mutated copies of the problematic genomic regions is usually possible, which in turn allows the recovery of the unmutated sequences from several independently mutated ones. However, the original SAM protocol relies on molecular cloning, which has a negative impact on the scalability of this approach when combined with high throughput sequencing technologies.

As a solution, we propose a new approach, NG-SAM, which relies solely on PCR and dilution steps, thus having more potential for automation and scaling–up. We built a pipeline performing realistic simulations of the NG-SAM protocol in order to assess its feasibility. We simulated two settings addressing different properties of the proposed protocol. The results of the first simulated setting raise the hope that by *in silico* and wetlab optimisation, it may be possible to find sets of experimental conditions by which NG-SAM is able to reconstruct most of the targets of interest which can be amplified through PCR. Moreover, the second simulation set suggests that these results are robust to variations in the all-important dilution factors.

Besides demonstrating the viability of the NG-SAM approach, the simulation pipeline can be useful in the future to aid in the design of actual experiments by allowing the selection of reasonable initial experimental conditions. It is also worth mentioning that the *de novo* assembly method used was successful in distinguishing and recovering a reasonable number of pooled mutant copies. Pooling many mutant types makes the assembly task harder, but to do so enhances the practicality of the proposed protocol. The number of libraries which can be barcoded and multiplexed on a single Illumina lane is limited. Pooling together fragments originating from distinct but related mutant types allows for a more efficient utilization of the coverage provided by the sequencing machines and also eliminates the difficult step of isolating individual mutant molecules.

As discussed below, NG-SAM is limited by the capabilities of PCR amplification; hence it is unlikely that the approach could benefit from more coverage than that provided by one Illumina lane. On the contrary, it would even be wasteful to spend the coverage provided by one lane on a single target region; however, this could be avoided by barcoding [26], which allows for using one lane for reconstructing multiple target regions or to perform NG-SAM experiments by making use of the “spare capacity" of a different experiment. Although we have used the Illumina platform in our simulations, the basic idea behind NG-SAM is not dependent on the sequencing technology and it should be straightforward to adapt it for other short read sequencing platforms such as Ion Torrent, which could offer further increase in cost effectiveness. Also, a straightforward way to increase the performance of the approach would be to use a short read platform offering longer read lengths, such as the recently–announced Illumina MiSeq.

The SAM approach is conceptually simple and appealing, and in principle could be used even to sequence microsatellites and other low complexity regions. The proposed NG-SAM protocol removes an important factor hindering scalability (molecular cloning) but there are still other factors limiting its use it in practice. These arise because of difficulties in performing PCR amplification of large DNA fragments. This is of particular concern in the case of the first, mutagenic, PCR but we believe that by using an enzymatic mixture containing a mutagenic polymerase and a polymerase with high processivity the mutagenesis of a fragment of length 10–15 kb should be possible. This would enable NG-SAM to target a significant portion of structural variations of interest, although this may need sophisticated laboratory optimization.

NG-SAM requires the knowledge of the sequence portions flanking the target region required for designing the primers used in the PCR reactions. There might be example use cases – e.g. large gaps in genomic sequence – where the approach cannot be used due to the absence of this information. Nevertheless, we believe that our approach has the potential to address many highly relevant use cases. These include, for instance, filling gaps in the human reference genome due to the tandem duplications with high sequence identity, an example where the knowledge about the flanking regions is available to enable the design of primers.

The other factor is the cost associated with performing “few molecule" PCR (the third PCR in the NG-SAM protocol), which is in itself laborious and risky in terms of contamination. Nevertheless, this procedure is more scalable than molecular cloning due to the recent developments in microfluidics technologies which enable the manipulation of reactants in very small volumes and might offer a practical solution for performing single molecule PCR in a safe and high-throughput way [27]. Microfluidics platforms such as that marketed by RainDance Technologies are already used to perform single molecule PCR experiments to amplify short fragments on a large scale as a part of NGS library construction procedures [28].

### Conclusions

Based on our simulated experiments, we conclude that under carefully chosen experimental conditions, the proposed NG-SAM approach based on PCR and dilution steps coupled with NGS technologies might be successfully put into practice. Having the practical hindrances removed, we believe that the NG-SAM approach has the potential to yield sequences as long as the emerging third generation sequencing platforms with the accuracy of established short read technologies.

## Materials and Methods

In the following sections we describe in detail the components of the simulation pipeline summarized in Figure 2. The most important parameters are summarized in Table 1. In accordance with the MIASE guidelines [29], the simulation pipeline has been made available online [30].

### Simulating PCR

The simulated experimental set-up involves three PCR steps (Figure 2): two are part of the mutagenic PCR protocol and a third provides coverage for short read sequencing. Although the forward simulation of PCR experiments is conceptually simple, it is not practical to carry out with a large number of starting molecules and many cycles: the number of molecules to keep track of increases exponentially and eventually requires too many resources in terms of CPU time and memory usage.

Fortunately our design does not require the simulation of every replication event, just those that involve one of the molecules that will be sampled after the second PCR or their “ancestors", and the total number of molecules after amplification. Thus, we were able to use the coalescent-based ‘backward’ simulation method developed by Weiss and von Haeseler [31], which we re-implemented in the pcrcoal R package [32]. This approach simulates a coalescent genealogy of the sampled molecules conditional on the number of starting molecules and the per-cycle PCR efficiencies. The sampled genealogy accounts for the relatedness of the sampled molecules, the branch lengths being the number of replications occurring on the respective lineages.

In all of the simulated PCR experiments we set the per-cycle efficiencies to 0.75, in line with previously published estimates [31]. In the case of the first (mutagenic) PCR and the second (cleanup) PCR we simulated 20 cycles (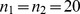 in Figure 2). We simulated 30 cycles in the case of the final PCR (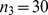).

### Simulating Molecule Sampling by Dilution

The sampling of molecules by dilution was modelled by taking random samples from Poisson distributions [33] with a mean equal to the expected number of molecules after dilution (the number of molecules in the original solution divided by the dilution factor). The simulated dilution factors were 70,000 for the first dilution (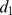; 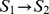 transition in Figure 2) and 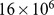 for the second dilution (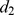; 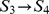 transition) in the first simulation setting. In the second simulation setting a range of dilution factors was explored as described in the Results section.

### Simulating Mutagenic PCR

In the first (mutagenic) PCR step we had to simulate the mutation intensity and spectrum in addition to accounting for the potential relatedness of the sampled molecules. The mutation spectrum (Figure S5A) produced by the mutagenic protocol has been reported [22] and we constructed a general non-reversible (UNREST) substitution model [34] to match this (Figure S5B), leaving only the rate of mutation per cycle of PCR to be calibrated.

We simulated many mutagenesis experiments using our model, starting with the same sequence as that on which the mutation spectrum was observed, and then picked a branch length scaling factor in order to match the reported average of 5% mutations after 10 cycles of mutagenic PCR [22]. This calibration procedure makes the assumption that no mutations are introduced during the cleanup PCR. Mutations on the genealogies were simulated under the inferred scaling factor and the general non-reversible substitution process constructed as described above.

The molecules sampled by dilution from the first PCR serve as initial molecules for the second PCR (Figure 2). The number of mutant types after the second dilution (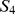) has an upper bound much smaller than the number of molecules sampled by the first dilution (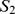). To save computing time we restricted the simulation of the genealogy to a sample size which is likely to be greater than the number of the molecules sampled after the second dilution (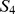), rather than simulating the full genealogy of all molecules present after amplification. As we assume that there are no mutations introduced in the second PCR, there was no need to simulate the genealogy in this case, just the number of molecules after amplification.

We used 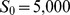 identical template molecules as an input for simulating mutagenic PCR reactions using the method described above.

### Simulating Illumina Sequencing

The molecules sampled after the second PCR serve as templates for the final PCR (Figure 2). We simulated sequencing with a total coverage of 4,000x, which provides approximately a 200x coverage per mutant type. This level of coverage can be easily obtained on one lane of an Illumina HiSeq sequencer for all simulated target region sizes. The exact coverage of the individual mutant types is proportional to the number of their descendants after the final PCR; thus we take into account the fluctuation in coverage due to the amplification.

We simulated Illumina sequencing by using the simNGS package version 1.5 [35], with default parameters and trained on intensity data produced by a paired-end run with a read length of 101 on an Illumina HiSeq sequencer. The simNGS package simulates the construction of sequencing libraries by random fragmentation as well as the ligation of adapter sequences. The size of the simulated fragments was sampled from a log-normal distribution with reasonable parameters: a mean of 400 (implying a mean insert size of 198) and a coefficient of variation of 0.055.

### Reconstructing the Consensus

We used Velvet [8] to assemble the simulated reads (development version aeb11f80). We instructed Velvet to estimate the expected coverage from the data (-exp_cov auto), and set the minimum contig length to 400. We used a long *k*-mer length (90) in order to increase the accuracy of the assembly on the expense of required coverage. We set the -max_divergence parameter to 0.1, preventing the simplification of “bubbles" in the assembly graph formed by sequences more diverged than 10%, in order to facilitate the recovery of the mutant types as individual contigs.

We used exonerate [36] v2.2.0 (with parameters -m affine:local -e −100 -o −100) to align the contigs to the longest one, and reverse complemented them to correct differences in orientation if necessary, used muscle [37] version 3.8.31 (with parameters: -maxiters 1 -diags 2) to perform multiple sequence alignment. We used the resulting alignment to call a “majority vote" consensus and aligned the consensus to the original target region using exonerate (parameters as above) in order to assess the accuracy of the reconstruction.

## Supporting Information

Figure S1
**Distribution of the number of molecules in the simulated samples after the second dilution (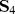) from all simulated experiments in the first setting.**
(TIFF)

Figure S2 **Performance of NG-SAM in the first simulation setting.** The hexagons are colored according to the mean of the metrics from all covered simulated experiments. White areas represent unexplored parameter space. The black circles at (3813, 3) mark the repetitive structure of the target region used in the second simulation setting. The dashed lines corresponds to target regions with a total size of 10 kb. **A**. The percentage of successful simulated experiments, as a function of the length and number of repetitive units in the target sequence. **B**. Percentage of correctly reconstructed bases in successful experiments, as a function of the length and number of repetitive units in the target sequence (a product of percentage sequence identity with respect to the target sequence – **C** – and proportion of recovered target sequence length – **D**). **C**. Percentage sequence identity with respect to the target sequence in successful experiments, as a function of the length and number of repetitive units. **D**. Percentage recovered sequence length in successful experiments as, a function of the length and number of repetitive units in the target sequence.
(TIFF)

Figure S3 **Schematic representation of the repetitive structure of the eater target region as a dot plot.** The dot plot of a single the *D. melanogaster eater* sequence against itself was constructed with the dotPlot method from the seqinr R package (http://cran.r-project.org/web/packages/seqinr) with parameters: wsize = 4, wstep = 4, nmatch = 4.
(TIFF)

Figure S4 **Performance of NG-SAM in the second simulation setting.** The hexagons are colored according to the mean of the metrics from all covered simulated experiments. The black circles corresponds to the dilution factors used in the first simulation setting. **A**. The percentage of successful simulated experiments, as a function of the dilution factors. **B**. Percentage of correctly reconstructed bases in successful experiments, as a function of the dilution factors (a product of percentage sequence identity with respect to the target sequence – **C** – and proportion of recovered target sequence length – **D**). **C**. Percentage sequence identity with respect to the target sequence in successful experiments, as a function of the dilution factors. **D**. Percentage recovered sequence length in successful experiments, as a function of the dilution factors.
(TIFF)

Figure S5 **Properties of the mutation process used in the NG-SAM simulations.** **A.** The mutation spectrum observed in the mutagenic PCR experiments performed by Zaccolo et al. (J. Mol. Biol., 1996). **B.** A “bubble plot" of the general non-reversible (UNREST) substitution process used in the NG-SAM simulations, constructed using the mutation spectrum shown in **A**.
(TIFF)

## References

[1] Metzker M (2010) Sequencing technologies - the next generation. Nat Rev Genet 11: 31–46. 19997069 10.1038/nrg2626

[2] Treangen T, Salzberg S (2011) Repetitive dna and next-generation sequencing: computational challenges and solutions. Nat Rev Genet Published online 29 November. 10.1038/nrg3117 PMC3324860 22124482

[3] Flicek P, Birney E (2009) Sense from sequence reads: methods for alignment and assembly. Nat Methods 6: S6–S12. 19844229 10.1038/nmeth.1376

[4] Garber M, Zody M, Arachchi H, Berlin A, Gnerre S, et al (2009) Closing gaps in the human genome using sequencing by synthesis. Genome Biol 10: R60. 19490611 10.1186/gb-2009-10-6-r60 PMC2718494

[5] International Human Genome Sequencing Consortium (2004) Finishing the euchromatic sequence of the human genome. Nature 431: 931–945. 15496913 10.1038/nature03001

[6] Mills RE, Walter K, Stewart C, Handsaker RE, Chen K, et al (2011) Mapping copy number variation by population-scale genome sequencing. Nature 470: 59–65. 21293372 10.1038/nature09708 PMC3077050

[7] Miller J, Koren S, Sutton G (2010) Assembly algorithms for next-generation sequencing data. Genomics 95: 315–327. 20211242 10.1016/j.ygeno.2010.03.001 PMC2874646

[8] Zerbino D, Birney E (2008) Velvet: algorithms for de novo short read assembly using de bruijn graphs. Genome Res 18: 821–829. 18349386 10.1101/gr.074492.107 PMC2336801

[9] Paszkiewicz K, Studholme D (2010) De novo assembly of short sequence reads. Brief Bioinform 11: 457–472. 20724458 10.1093/bib/bbq020

[10] Wessler S (2006) Transposable elements and the evolution of eukaryotic genomes. PNAS 103: 1760–1760. 10.1073/pnas.0607612103 PMC1693792 17101965

[11] Zhang J (2003) Evolution by gene duplication: an update. Trends in Ecology & Evolution 18: 292–298.

[12] Nielsen R, Paul J, Albrechtsen A, Song Y (2011) Genotype and snp calling from next-generation sequencing data. Nature Reviews Genetics 12: 443–451. 10.1038/nrg2986 PMC3593722 21587300

[13] Medvedev P, Stanciu M, Brudno M (2009) Computational methods for discovering structural variation with next-generation sequencing. Nat Methods 6: S13–20. 19844226 10.1038/nmeth.1374

[14] Bailey J, Eichler E (2006) Primate segmental duplications: crucibles of evolution, diversity and disease. Nat Rev Genet 7: 552–564. 16770338 10.1038/nrg1895

[15] Liu S, Yao L, Ding D, Zhu H (2010) CCL3L1 copy number variation and susceptibility to HIV-1 infection: a meta-analysis. PLoS One 5: e15778. 21209899 10.1371/journal.pone.0015778 PMC3012711

[16] Zerbino D, McEwen G, Margulies E, Birney E (2009) Pebble and rock band: heuristic resolution of repeats and scaffolding in the velvet short-read de novo assembler. PLoS One 4: e8407. 20027311 10.1371/journal.pone.0008407 PMC2793427

[17] Keith J, Cochran D, Lala G, Adams P, Bryant D, et al (2004) Unlocking hidden genomic sequence. Nucleic Acids Res 32: e35. 14973330 10.1093/nar/gnh022 PMC373418

[18] Mitchelson K (2011) Sequencing of difficult DNA regions by SAM sequencing. Methods Mol Biol 687: 75–88. 20967602 10.1007/978-1-60761-944-4_6

[19] Keith J, Hawkes D, Carter J, Cochran D, Adams P, et al.. (2007) Sequencing aided by mutagenesis facilitates the de novo sequencing of megabase DNA fragments by short read lengths. In: Mitchelson KR, editor, New High Throughput Technologies for DNA Sequencing and Genomics, Elsevier, volume 2 of Perspectives in Bioanalysis. 303–326. doi: 10.1016/S1871–0069(06)02010–6.

[20] Keith J, Adams P, Bryant D, Kroese D, Mitchelson K, et al (2002) A simulated annealing algorithm for finding consensus sequences. Bioinformatics 18: 1494–1499. 12424121 10.1093/bioinformatics/18.11.1494

[21] Keith J, Adams P, Bryant D, Cochran D, Lala G, et al (2004) Algorithms for sequence analysis via mutagenesis. Bioinformatics 20: 2401–2410. 15145816 10.1093/bioinformatics/bth258

[22] Zaccolo M, Williams D, Brown D, Gherardi E (1996) An approach to random mutagenesis of DNA using mixtures of triphosphate derivatives of nucleoside analogues. J Mol Biol 255: 589–603. 8568899 10.1006/jmbi.1996.0049

[23] JBS dNTP-Mutagenesis Kit; Accessed 11 jul 2012. URL http://www.jenabioscience.com/images/0ea5cbe470/PP-101.pdf.

[24] Kocks C, Cho JH, Nehme N, Ulvila J, Pearson AM, et al (2005) Eater, a transmembrane protein mediating phagocytosis of bacterial pathogens in Drosophila. Cell 123: 335–346. 16239149 10.1016/j.cell.2005.08.034

[25] Somogyi K, Sipos B, Pénzes Z, Kurucz É, Zsámboki J, et al (2008) Evolution of genes and repeats in the Nimrod superfamily. Mol Biol Evol 25: 2337–2347. 18703524 10.1093/molbev/msn180

[26] Meyer M, Kircher M (2010) Illumina sequencing library preparation for highly multiplexed target capture and sequencing. Cold Spring Harb Protoc 2010: pdb.prot5448. 20516186 10.1101/pdb.prot5448

[27] Tewhey R, Warner J, Nakano M, Libby B, Medkova M, et al (2009) Microdroplet-based PCR enrichment for large-scale targeted sequencing. Nat Biotechnol 27: 1025–1031. 19881494 10.1038/nbt.1583 PMC2779736

[28] Blow N (2009) Genomics: catch me if you can. Nature Methods 6: 539–544.

[29] Waltemath D, Adams R, Beard DA, Bergmann FT, Bhalla US, et al (2011) Minimum information about a simulation experiment (MIASE). PLoS Comput Biol 7: e1001122. 21552546 10.1371/journal.pcbi.1001122 PMC3084216

[30] The NG-SAM simulation pipeline; Accessed 11 jul 2012. URL https://github.com/sbotond/paper-ng-sam.

[31] Weiss G, von Haeseler A (1997) A coalescent approach to the polymerase chain reaction. Nucleic Acids Res 25: 3082–3087. 9224608 10.1093/nar/25.15.3082 PMC146862

[32] The pcrcoal package; Accessed 11 jul 2012. URL http://cran.r-project.org/web/packages/pcrcoal.

[33] Stallard N, Gravenor M, Curnow R (2006) Estimating numbers of infectious units from serial dilution assays. Journal of the Royal Statistical Society: Series C (Applied Statistics) 55: 15–30.

[34] Yang Z (1994) Estimating the pattern of nucleotide substitution. J Mol Evol 39: 105–111. 8064867 10.1007/BF00178256

[35] simNGS and simlibrary – software for simulating next-gen sequencing data; Accessed 11 jul 2012. URL http://www.ebi.ac.uk/goldman-srv/simNGS/.

[36] Slater G, Birney E (2005) Automated generation of heuristics for biological sequence comparison. BMC Bioinformatics 6: 31. 15713233 10.1186/1471-2105-6-31 PMC553969

[37] Edgar R (2004) MUSCLE: a multiple sequence alignment method with reduced time and space complexity. BMC Bioinformatics 5: 113. 15318951 10.1186/1471-2105-5-113 PMC517706

