# Supplementary figures and images for: An Improved Protocol for Sequencing of Repetitive Genomic Regions and Structural Variations Using Mutagenesis and Next Generation Sequencing
Source: PLoS One. 2012 Aug 17;7(8):e43359. doi: 10.1371/journal.pone.0043359 (PMC3422288; doi:10.1371/journal.pone.0043359)

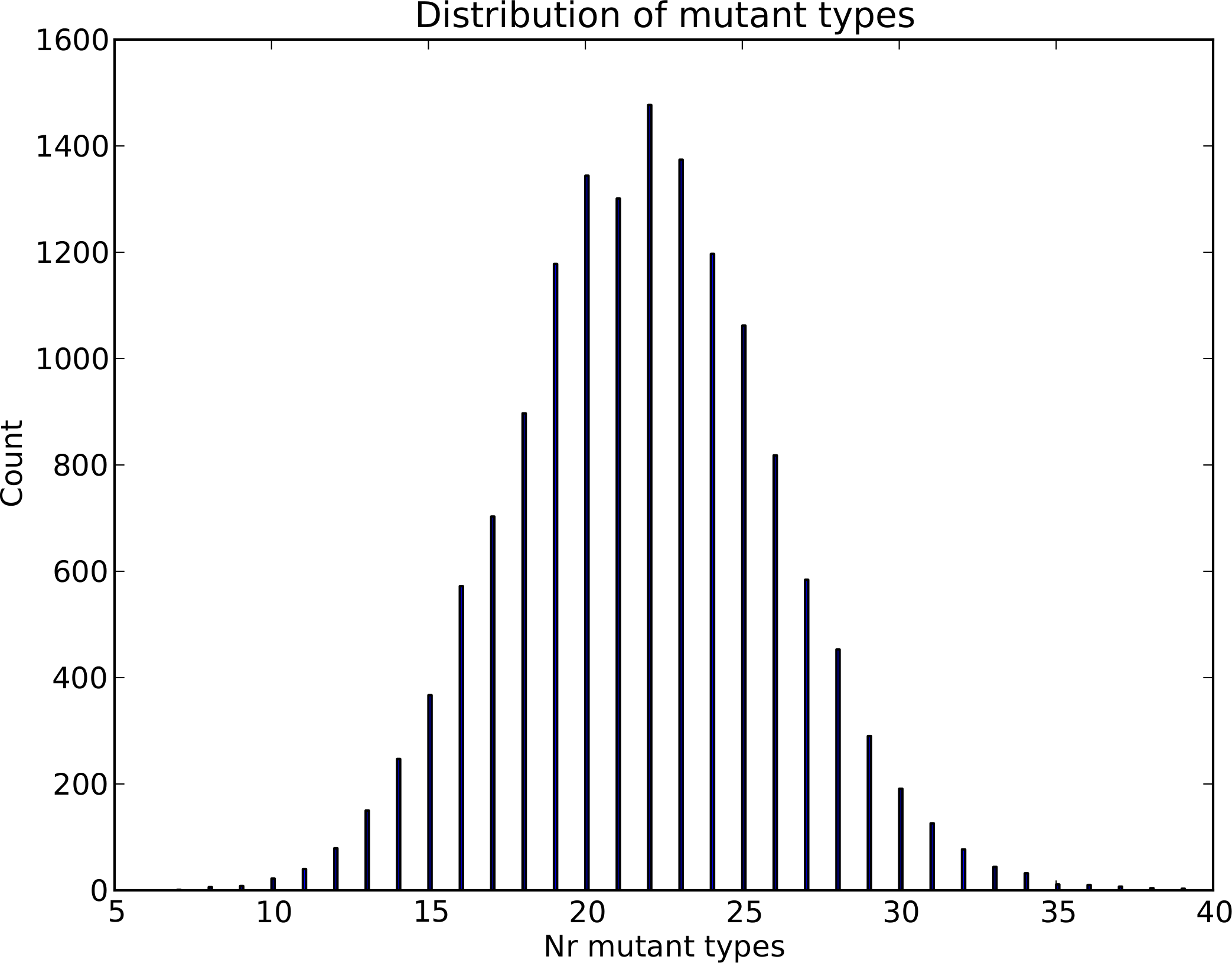

Supplement: Figure S1 — Distribution of the number of molecules in the simulated samples after the second dilution () from all simulated experiments in the first setting. (TIFF) [file pone.0043359.s001.tiff]

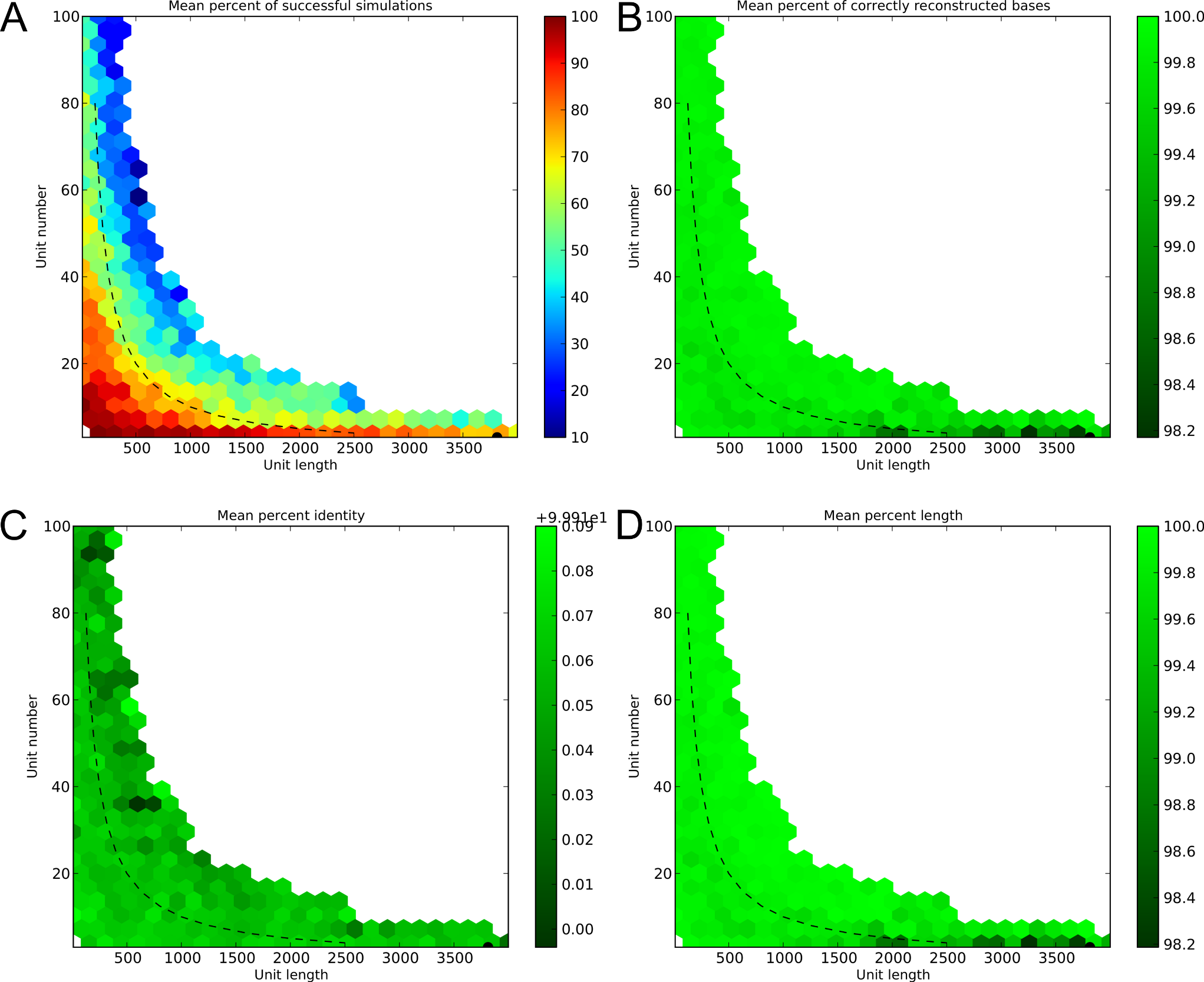

Supplement: Figure S2 — Performance of NG-SAM in the first simulation setting. The hexagons are colored according to the mean of the metrics from all covered simulated experiments. White areas represent unexplored parameter space. The black circles at (3813, 3) mark the repetitive structure of the target region used in the second simulation setting. The dashed lines corresponds to target regions with a total size of 10 kb. A. The percentage of successful simulated experiments, as a function of the length and number of repetitive units in the target sequence. B. Percentage of correctly reconstructed bases in successful experiments, as a function of the length and number of repetitive units in the target sequence (a product of percentage sequence identity with respect to the target sequence – C – and proportion of recovered target sequence length – D). C. Percentage sequence identity with respect to the target sequence in successful experiments, as a function of the length and number of repetitive units. D. Percentage recovered sequence length in successful experiments as, a function of the length and number of repetitive units in the target sequence. (TIFF) [file pone.0043359.s002.tiff]

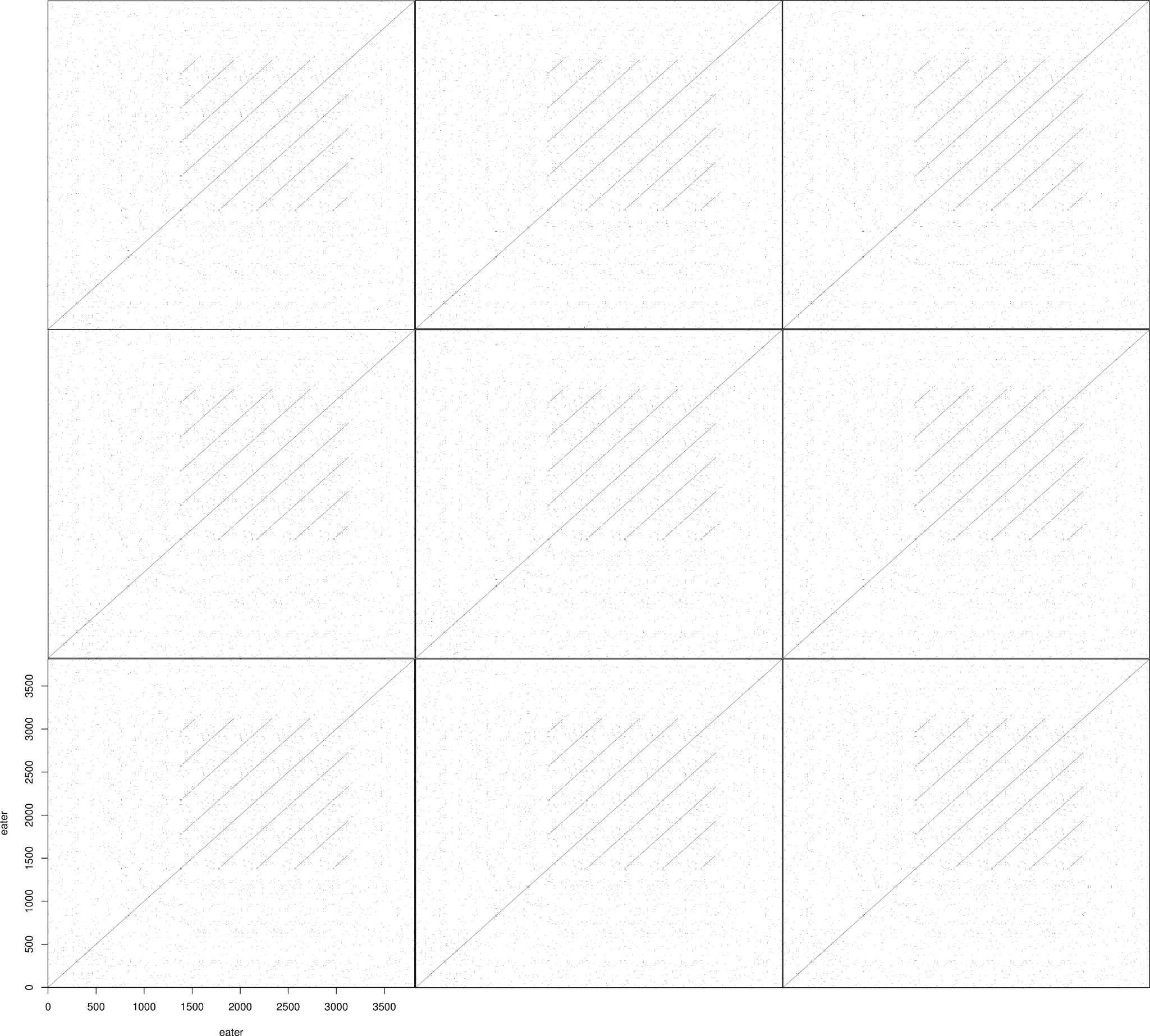

Supplement: Figure S3 — Schematic representation of the repetitive structure of the eater target region as a dot plot. The dot plot of a single the D. melanogaster eater sequence against itself was constructed with the dotPlot method from the seqinr R package (http://cran.r-project.org/web/packages/seqinr) with parameters: wsize = 4, wstep = 4, nmatch = 4. (TIFF) [file pone.0043359.s003.tiff]

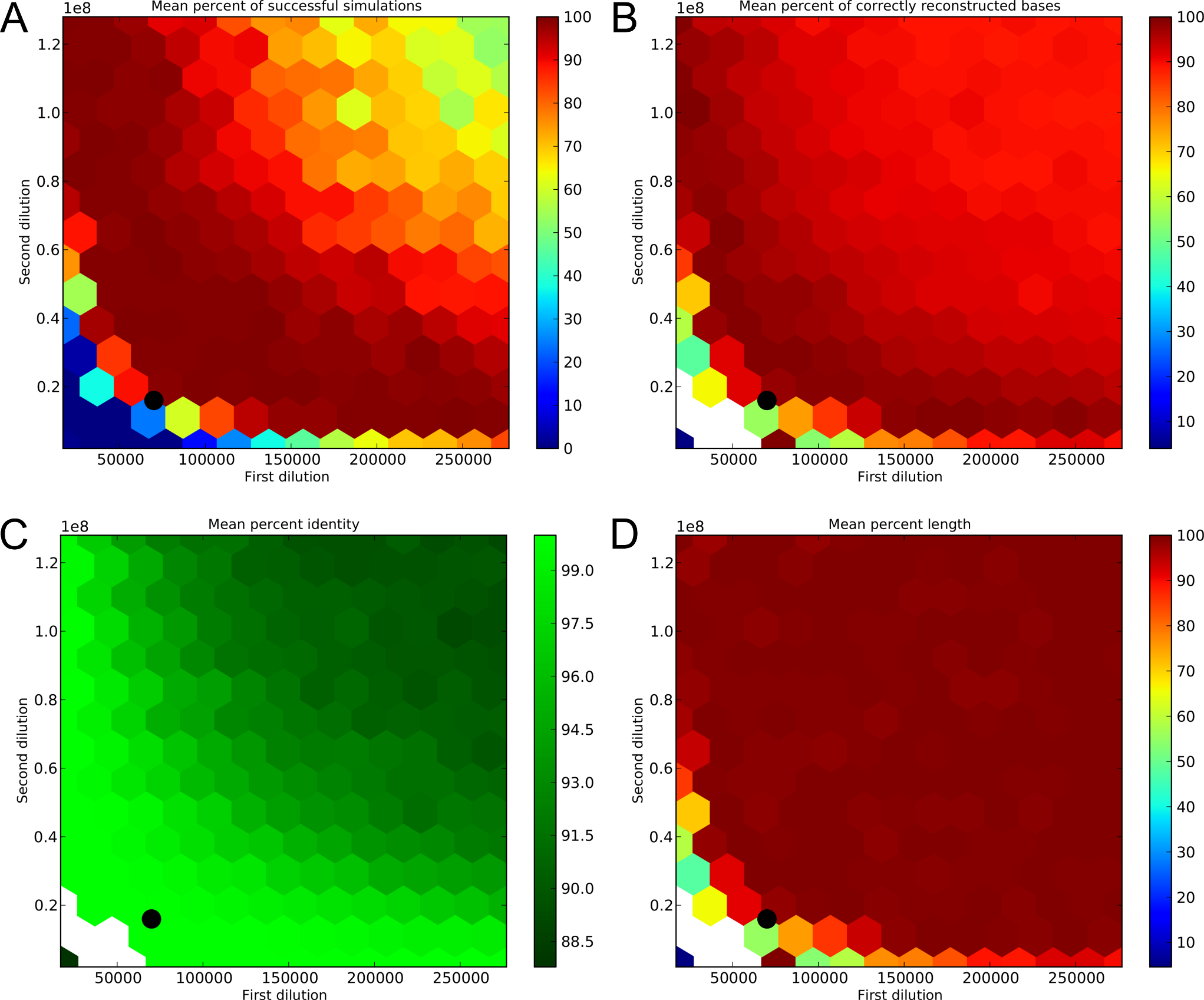

Supplement: Figure S4 — Performance of NG-SAM in the second simulation setting. The hexagons are colored according to the mean of the metrics from all covered simulated experiments. The black circles corresponds to the dilution factors used in the first simulation setting. A. The percentage of successful simulated experiments, as a function of the dilution factors. B. Percentage of correctly reconstructed bases in successful experiments, as a function of the dilution factors (a product of percentage sequence identity with respect to the target sequence – C – and proportion of recovered target sequence length – D). C. Percentage sequence identity with respect to the target sequence in successful experiments, as a function of the dilution factors. D. Percentage recovered sequence length in successful experiments, as a function of the dilution factors. (TIFF) [file pone.0043359.s004.tiff]

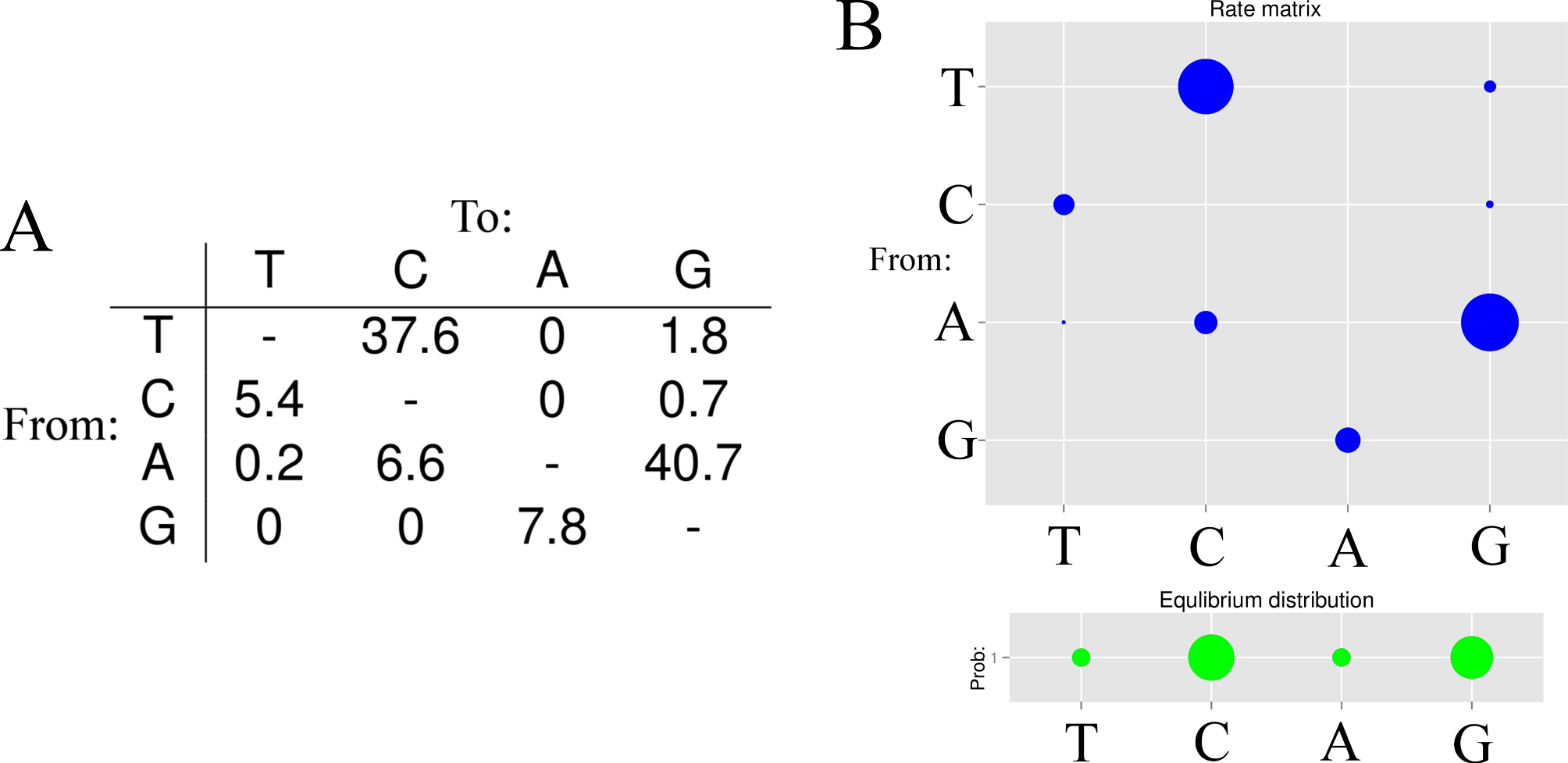

Supplement: Figure S5 — Properties of the mutation process used in the NG-SAM simulations. A. The mutation spectrum observed in the mutagenic PCR experiments performed by Zaccolo et al. (J. Mol. Biol., 1996). B. A “bubble plot" of the general non-reversible (UNREST) substitution process used in the NG-SAM simulations, constructed using the mutation spectrum shown in A. (TIFF) [file pone.0043359.s005.tiff]
